# Retrospective introspection

**DOI:** 10.4103/0019-5545.49446

**Published:** 2009

**Authors:** M. Thirunavukarasu, Pragatheeshwar Thirunavukarasu

**Affiliations:** Department of Psychiatry, Stanley Medical College, Member of Senate and President Faculty of Medical Specialties, The Tamil Nadu Dr. MGR Medical University, Chennai, India; 1 University of Pittsburgh Medical Center, Pittsburgh, USA

The following is a dialogue from Plato's famous work, ‘Republic’

Socrates: *Shall we set down astronomy among the subjects of study?* Glaucon: *I think so, to know something about the seasons, the months and the years is of use for military purposes, as well as for agriculture and for navigation.* Socrates: *It amuses me to see how afraid you are, lest the people should accuse you of recommending useless studies.*

We are recently faced with the tremendous task of formatting a system of teaching and training undergraduate and postgraduate students, the science and art of psychiatry. I would like to lay down the specific dilemma that faces us today – Should the curriculum in psychiatry training involve basic science education namely – neuroanatomy, neuroscience, neurophysiology, clinical neurology, psychology, psychopharmacology, etc and the henceforth evaluation of such education be done by specialists and experts in such fields or should the curriculum in psychiatry involve exclusively applied and problem-based basic science education in the above mentioned subjects and the henceforth evaluation of education be done by academic psychiatrists themselves?

The analysis of this issue pulls us back to a question that has been largely debated in academic circles, especially in the area of basic science education (anatomy, physiology, biochemistry, etc) for medical students. The two broad approaches to basic science education are

**Traditional learning method –** often called the *‘basic science’* method; a comprehensive, strong education in basic science principles followed by modules to achieve application of those principles in field practice.

**Problem-based learning method –** often called the *‘applied science’* method; wherein there is specific focus only on the aspects of basic sciences that are applied in the clinical setting.

## PROBLEM-BASED LEARNING

Problem based learning (PBL) is a philosophy that promotes the idea that the teaching of a certain principle should be done by posing a problem, the solution of which can be reached by the knowledge of that principle. Introduced in the middle half of the 20^th^ century, PBL started in North America and quickly gained unexpected popularity in schools, colleges and universities. PBL often involves small group discussions, as opposed to the conventional lecture based curriculum and often involved immediate feedback from the group.

As this approach received unprecedented welcome and support, it slowly evolved to a situation where the utility of basic science knowledge in the ‘field practice’ came to be questioned. Also, there was a flurry of studies that showed that students evaluated and assessed themselves to be more competent and confident in their educational results in the PBL technique.

This trend also led to the adoption and modification of medical education curriculum that involved more ‘application-based’ teaching of concepts, thereby reducing the effort and time spent on pure basic science education.

However, the pendulum now seems to swing in the opposite direction. Albanese *et al*,[1] in 1993 published a meta-analysis type review of the English international literature between 1972 and 1992. The findings that and such similar studies can be summarized as follows:

- PBL is more nurturing and enjoyable for the students
- PBL is more enjoyable for the teaching faculty
- PBL students performed similar to conventionally taught students in clinical evaluations
- PBL students seemed to engage in ‘backward’ reasoning as opposed to ‘forward’ reasoning, which is what experts engage in
- PBL students display ‘gaps’ in cognitive knowledge ‘weaknesses’ in cognitive processing that affects practice outcome
- PBL techniques are expensive and are high resource utilizing methods

We don't know enough about PBL, and hence widespread embracement of this technique should be done with caution

Chang *et al*,[2] analyzed the effect of PBL techniques in surgical education and concluded that, “*Centers that have adopted a PBL approach have found improved student motivation and enjoyment, but there has been no convincing evidence of improved learning*”

The above findings are not surprising to the astute mind. While PBL and hence applied science teaching and the henceforth evaluation by the proponents of the same philosophy can provide higher levels of comfort and a ‘feel-good’ effect, this never translates into better clinical practice.

What is more frightening is the ‘cognitive gap’ in the applied science teaching methods. Such cognitive gaps are due to the above mentioned *‘backward reasoning’*[3] employed by PBL students i.e. from clinical information to theory rather than the *‘forward reasoning’* employed by conventional students i.e. theory to practice. Such cognitive gaps and weaknesses can lead to disastrous long term effects in the presence of euphoric effect it gives.

## BASIC VS. APPLIED SCIENCE

While the real value of PBL and applied science teaching methods are yet to be analyzed by well-designed randomized trials, it seems obvious that such teaching techniques are not the gold standard. Common sense would mandate that any fruitful application of basic science will not occur without the strong fundamental basis of the pure basic science itself. So, where does this enthusiasm for applied science learning come from? It is worth analyzing the evolution of thought in this field. Let's make some definitions, to avoid ambiguities –

**Basic science –** motivated by *curiosity*, regardless of application. E.g.: Why is the sky blue? Where is the sun? Why do we have fever?

**Applied science –** motivated by *industry*, designed to answer specific questions. E.g.: How shall we make a faster aircraft? How can I treat fever?

The traditional belief is the “linear model” – wherein basic science education and research is supposed to lead to a specific application that leads to profit in industry or a benefit in clinical practice. This has been largely true; however, it is easy to find examples for the opposite, wherein we have learnt more science from specific innovations. One such example is what George Porter (Nobel Laureate in Chemistry) said – *“Thermodynamics owes more to the steam engine than the steam engine owes to science”*. Such popular occurrences lead to people strongly advocating the “anti-linear model” and hence the advocacy of the ‘learn-only-what-is-necessary’ policy. Truthfully, the philosophy of science and its application is neither linear nor ant-linear, but ‘*non-linear*’ – meaning that the integration of basic knowledge and its application is inseparable.

Llewellyn Smith,[4] the Director-General of CERN notes, “*I do not like the terms basic and applied science: after all who can say in advance what is applicable?*” He further notes that the definitions of the terms are simply based on ‘motivation’ that drives the study of the science. He also explains his stand by using the example of J. J. Thomson,[5] the discoverer of electron, who argued that the use of X-rays in medicine, (which became popular during war, wherein they were used to locate bullets in gun-shot victims) resulted from the pure basic science approach towards metallurgy and electricity that resulted in an application in the industry and not the result of an applied science approach – “how to locate bullets in human bodies?”, which may have only resulted in improved probing techniques in wounds. J.J Thompson also went on to imply that *while applied science leads to improvements in old methods, it is basic science that leads to new methods*.

However, it is undeniable that basic and applied sciences are better understood together, rather than separate. Together, they function better and complement each other. Interesting, some medical schools in the USA are attempting to re-introduce basic sciences *‘again’* in final year of medical school,[6] as this not only strengthens their understanding of clinical medicine, but also enhances the understanding and importance of basic sciences.

## EDUCATION OF PSYCHIATRY AS A *‘SCIENTIFIC’* DISCIPLINE

Psychiatry has been a scientific discipline, lauded in medicine, for more than a century. Psychiatry is attractive to several medical students and is being increasingly eyed with interest by the cream of the undergraduates. At this juncture, it is very essential for the academic community and faculty to strongly hold on to the authentic and time-tested methods of education. Living in such interesting and crucial times, it is important that we act responsibly, yet definitively.

From the first author's 30 odd years in psychiatry which includes extensive experience in training medical students, postgraduates, nurses, allied medical specialists and communicating with the people in a large scale through the media, it is my humble yet deliberate opinion and belief that *psychiatry needs to be approached, taught, learnt, researched and practiced on a fully scientific basis with scientifically and logically based changes made to its curriculum with regard to our society, culture, history and heritage.*

The ultimate and often misleading rebuttal to the above idea comes from people who exemplify the western psychiatric academic institutions which have adopted applied science, PBL techniques and internal review evaluations. Such examples are rudimentary and unscientific and it is essential to understand the following, before making such sweeping rebuttals

The western institutions have other mechanisms and systems in place which buffer the deficiencies that may result from an applied science approach. Such mechanisms may be absent in our institutional model that may expose us to deleterious effects if such deficiencies if it were to happen to us. Examples include a higher exposure to clinical psychiatry in medical school training, examination and evaluation of theoretical and clinical skills in psychiatry in undergraduate training, etc.

A *default unproven assumption and belief* that western institution model has better learning and teaching techniques compared to Indian educational systems.

An attempt to ape the western institutions' model without any scientific basis, especially in the presence of evidence supporting the contrary, defeats virtues of common sense and self-respect.

Regardless, we may just be an unfertile soil for the westernized model. As an analogy – the oak tree that flourishes in the foreign soil would wither away in our native soil, where palm trees may grow and flourish.

## PROPOSED MODEL

I hereby propose the following model of education and evaluation in postgraduate psychiatry –

- A strong education of the fundamental principles of the psychiatric basic sciences namely, neuroanatomy, neurophysiology, neurochemistry, psychology, behavioral sciences, neuroimaging, genetics and psychopharmacology. The teaching and evaluation must preferably be done by qualified experts from the respective field, who understand the practical limitations of interdisciplinary learning
- A separate focus on methods of statistics and research, which will enable trainees to logically inquire and critically analyzed published data.
- A revisiting of basic science subjects at the end of the first clinical year to reinforce understanding of basic sciences and clinical practices.
- I open the above model to opinions, suggestions and constructive criticisms from all those who wish a strong education for the young vibrant minds that look forward for a career and life in psychiatry.
